# Second Covered and Uncovered Self-Expandable Metal Stents for Recurrent Gastric Outlet Obstruction: A Retrospective Comparative Study

**DOI:** 10.3390/jcm12165241

**Published:** 2023-08-11

**Authors:** Naminatsu Takahara, Yousuke Nakai, Kota Ishida, Go Endo, Kohei Kurihara, Shuichi Tange, Shinya Takaoka, Yurie Tokito, Yukari Suzuki, Hiroki Oyama, Sachiko Kanai, Tatsunori Suzuki, Tatsuya Sato, Ryunosuke Hakuta, Kazunaga Ishigaki, Tomotaka Saito, Tsuyoshi Hamada, Mitsuhiro Fujishiro

**Affiliations:** 1 Department of Gastroenterology, Graduate School of Medicine, The University of Tokyo, Tokyo 113-8655, Japan; naminatsu-takahara@umin.ac.jp (N.T.); kot.ishida@gmail.com (K.I.); g.endo.1213@gmail.com (G.E.); kuriharak-int@h.u-tokyo.ac.jp (K.K.); tanges-int@h.u-tokyo.ac.jp (S.T.); takaokas-int@h.u-tokyo.ac.jp (S.T.); tokitoy-int@h.u-tokyo.ac.jp (Y.T.); yukasuzuki.tuk@gmail.com (Y.S.); hiroki.o2.1004@gmail.com (H.O.); sachiko.kanai.419@gmail.com (S.K.); suzutatsu86@gmail.com (T.S.); satyu12@gmail.com (T.S.); hakuta-tky@umin.ac.jp (R.H.); ishigakikazunaga@gmail.com (K.I.); tomsaito-gi@umin.ac.jp (T.S.); hamada-tky@umin.ac.jp (T.H.); mtfujish@gmail.com (M.F.); 2 Department of Endoscopy and Endoscopic Surgery, Graduate School of Medicine, The University of Tokyo, Tokyo 113-8655, Japan; 3 Department of Chemotherapy, The University of Tokyo Hospital, Tokyo 113-8655, Japan; 4 Department of Hepato-Biliary-Pancreatic Medicine, Cancer Institute Hospital, Japanese Foundation for Cancer Research, Tokyo 135-8550, Japan

**Keywords:** recurrent gastric outlet obstruction, second self-expandable metal stent, endoscopic stent placement

## Abstract

**Background:** Endoscopic self-expandable metal stent (SEMS) placement is a current mainstay for malignant gastric outlet obstruction (GOO), but symptomatic recurrence due to initial SEMS dysfunction commonly occurs. We aimed to compare the safety and effectiveness of second SEMS for recurrent GOO (RGOO). **Methods:** Between April 2006 and December 2022, a total of 95 cases with malignant RGOO undergoing second endoscopic SEMS placement were enrolled. Technical and clinical success rates, RGOO, time to RGOO (TRGOO), stent patency rate, adverse events (AE), and overall survival (OS) were retrospectively compared between covered and uncovered SEMS (cSEMS/uSEMS) groups. Risk factors for TRGOO were also explored. **Results:** Baseline characteristics were well balanced between cSEMS (*n* = 48) and uSEMS (*n* = 47) groups, except for the causes of the initial SEMS dysfunction. High technical and clinical success rates with a similar incidence of AE (15% vs. 17%, *p* = 0.78) and OS (median of 101 vs. 102 days, *p* = 0.68) were achieved in both groups. There were no statistical differences in cumulative incidence of RGOO (19% vs. 13%, *p* = 0.58), TRGOO (median, not reached in both groups, *p* = 0.57), and stent patency rates at 1, 2, and 3 months between the groups (60%, 47% and 26%, respectively vs. 70%, 55% and 38%, respectively). However, TRGOO tended to be longer in cSEMS in cases with RGOO due to tumor ingrowth (median, not reached vs. 111 days, *p* = 0.19). A Cox regression analysis demonstrated that chemotherapy after second SEMS placement was significantly associated with an improved TRGOO (the hazard ratio of 0.27 [95% confidence interval, 0.08–0.93], *p* = 0.03). **Conclusions:** Regardless of the type of SEMS, second SEMS placement was similarly safe and effective for RGOO. The type of second SEMS might be considered based on the cause of initial SEMS dysfunction.

## 1. Introduction

Malignant gastric outlet obstruction (GOO) can be caused by several types of cancer and is characterized by various uncomfortable symptoms such as nausea, postprandial vomiting, epigastric abdominal pain, and eventually leading to malnutrition, impaired quality of life and poor survival [1]. Endoscopic self-expandable metal stent (SEMS) placement has been widely accepted for [only unresectable cases] malignant GOO especially in cases with a short life expectancy (<3 months) because it offers rapid symptomatic relief with low morbidity and mortality as compared to surgical gastrojejunostomy [2,3,4,5]. However, the major drawback of endoscopic SEMS placement is an inherent risk of recurrent symptoms of GOO (RGOO) due to SEMS dysfunction requiring salvage intervention.

Covered SEMS (cSEMS) has been developed to minimize the risk of stent occlusion by tumor ingrowth, which is the major cause of uncovered SEMS (uSEMS) dysfunction [6,7,8]. However, this potential advantage of cSEMS can be offset by the increased risk of migration, resulting in an equivalent stent patency and reintervention rate between these two SEMSs [9,10]. With improving survival in various cancers owing to multidisciplinary approach, cases with malignant GOO are predisposed to RGOO and more likely to require reinterventions for SEMS dysfunction [11]. Several studies demonstrated second SEMS placement represents a minimally invasive and effective option for the management of RGOO caused by the initial SEMS dysfunction [12,13]. However, little is known about the selection of the SEMS type. 

Herein, we conducted this study to compare the safety and effectiveness of second cSEMS and uSEMS for RGOO and explore factors associated with time to RGOO (TRGOO) after second SEMS placement.

## 2. Methods

This is a single-center, retrospective study conducted at The University of Tokyo Hospital to compare the safety and effectiveness of second cSEMS and uSEMS for RGOO due to the initial SEMS dysfunction in cases with malignant GOO. Written informed consent for endoscopic interventions was obtained from all patients before the procedure. This study was approved by the ethics committee of the hospital, and consent for use of data for research was obtained on an opt-out basis.

### 2.1. Patients

**Figure 1:**
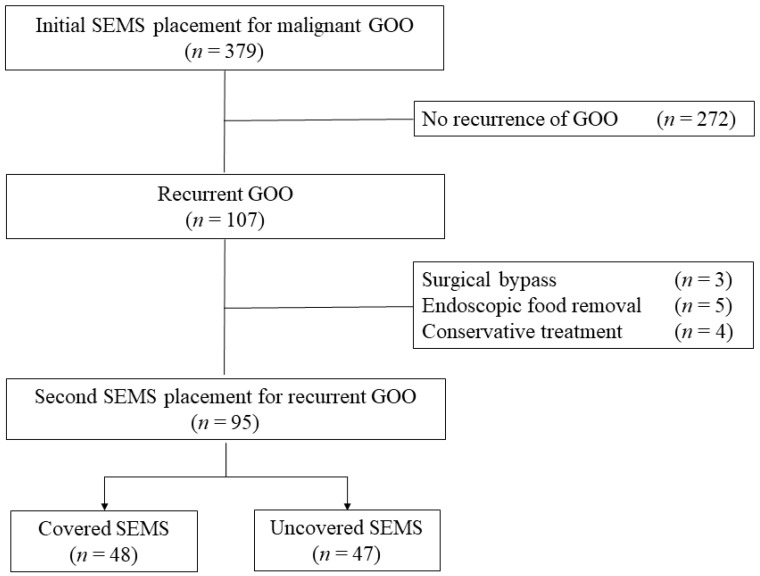
Patient flow. SEMS, self-expandable metal stent; GOO, gastric outlet obstruction.

Between April 2006 and December 2022, 379 cases that underwent endoscopic initial SEMS placement for symptomatic malignant GOO at The University of Tokyo Hospital were identified from our prospectively maintained database. Of these, 107 cases (28%) developed RGOO due to the initial SEMS dysfunction. After exclusion of patients who underwent surgical bypass (*n* = 3), endoscopic removal of impacted food (*n* = 5), and conservative treatment with parenteral nutrition (*n* = 4), a total of 95 patients undergoing second SEMS placement for the management of RGOO were enrolled in this study (Figure 1). 

### 2.2. Outcomes and Definitions

The primary outcome was cumulative time to recurrent GOO (TRGOO), and the secondary outcomes included technical and clinical success, the incidence of recurrent GOO (RGOO), adverse events, and overall survival (OS) after second SEMS placement. We additionally explored factors associated with TRGOO and investigated the outcomes of second SEMS according to the cause of initial SEMS dysfunction.

Technical success was defined as adequate placement of the SEMS across the stricture, as confirmed by a combination of endoscopy and fluoroscopy. Clinical success was defined as the improvement of the GOOSS score of at least one point within three days after SEMS placement and the ability of oral intake was evaluated based on the gastric outlet obstruction scoring system (GOOSS). RGOO was defined as the recurrence of symptoms associated with GOO such as nausea, vomiting, or abdominal distention, and the cause of RGOO was evaluated by computed tomography and/or an endoscopic examination when feasible. TRGOO was defined as time from stent placement to RGOO with any cause and was calculated only in patients who were able to resume oral intake after stent placement (GOOSS score of >2). OS was defined as the time from SEMS placement until death from any cause. The follow-up period was until the end of February 2023.

Adverse events were defined and graded according to the lexicon of the American Society for Gastrointestinal Endoscopy [14]. We regarded asymptomatic stent migration as an adverse event not as RGOO since it did not develop any symptoms related to GOO recurrence. 

### 2.3. Self-Expandable Metal Stents (SEMSs) and Stent Placement Procedure

**Figure 2:**
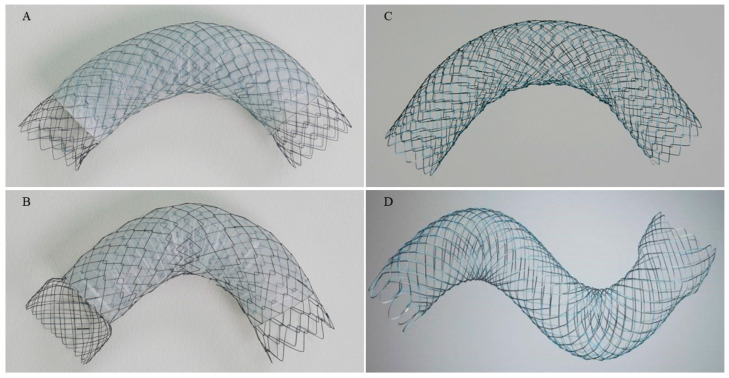
Self-expandable metal stent (**A**) ComVi stent (non-flared type); a partially covered stent with an uncovered non-flared proximal and distal end. The diameter of non-flared ComVi stent is all 22 mm and the length of 8, 10, 12 cm are available. (**B**) ComVi stent (flared type); a partially covered stent with an uncovered flared proximal and non-flared distal end. The diameter of flared ComVi stent is 20 mm and 24 mm and the lengths of 6, 9, 12, and 15 cm are available. (**C**) Niti-S is an uncovered stent without a flare end characterized by a low axial and radial force. The diameter of 22 mm and the lengths of 6, 8, 10, and 12 cm are available. (**D**) WallFlex is an uncovered stent with a proximal flare end characterized by a relatively high axial and radial force. The diameter of 22 mm and the lengths of 6, 9, and 12 cm are available.

The cSEMSs used in this study were ComVi stents (Taewoong Medical Co., Ltd., Gimpo, Republic of Korea), which were constructed of a polytetrafluoroethylene (PTFE) membrane sandwiched between two uncovered nitinol wires with an uncovered portion at both ends. As shown in Figure 2, ComVi stents included three subtypes according to the diameter and the shape of proximal uncovered end: 20 mm without a flare, 20 mm with a flare, and 24 mm with a flare. The stent is loaded into a 10.5 Fr delivery system and lengths of 8, 10, 12, and 15 cm were commercially available. The uSEMS included Niti-S pyloric/duodenal stent (Taewoong Medical Co., Ltd.) and WallFlex Duodenal stent (Boston Scientific Corporation, Natick, MA, USA). Both these two uSEMSs expand up to 22 mm. WallFlex Duodenal stent has a proximal flare of 25 mm while Niti-S pyloric/duodenal stent does not have a proximal flare. Stent lengths of 8, 10, and 12 cm were selected based on the length and location of the stricture. ComVi stent and Niti-S stent are characterized by a low axial force, which allows them to maintain their bent shape when placed in a tortuous portion of gastrointestinal tract, while WallFlex Duodenal stent had a high radial force that would open up the tight stricture as well as a high axial force that would straighten the stent. 

**Figure 3:**
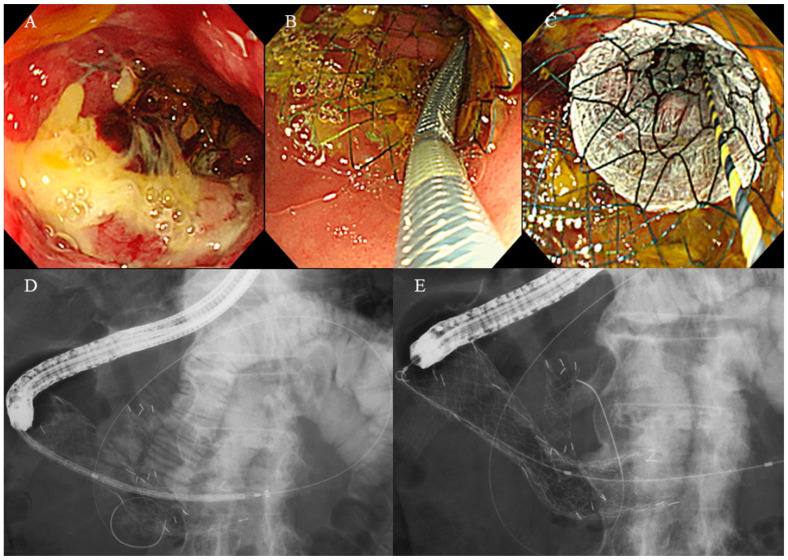
Stent placement procedure. (**A**) A guidewire was passed through the stricture under fluoroscopy. (**B**) A delivery system was positioned across the stricture. (**C**–**E**) Endoscopic and fluoroscopic view of a deployed 24 mm-ComVi stent.

As previously reported [15], second SEMS was placed by stent-in-stent technique using a therapeutic endoscope equipped with 3.7 or 4.2 mm accessory channel (Figure 3), except for cases with initial SEMS migration. A SEMS long enough to cover the stricture was selected in each case and was carefully deployed under endoscopic and fluoroscopic guidance. Second SEMS was chosen at the discretion of the attending endoscopist. While cSEMS was preferred in cases with RGOO due to tumor ingrowth, uSEMS was preferred in cases with RGOO due to stent migration and in cases with GOO involving the ampulla of Vater.

### 2.4. Statistical Analysis

Results [this study presented other results: e.g., TRGOO] were presented as number (%) or median (interquartile range [IQR]). Categorical variables were compared by using χ^2^ or Fisher’s exact tests. All continuous variables were not normally distributed and thus were compared using Mann–Whitney U test. Pre- and post-GOOSS scores were compared by using the Wilcoxon signed-rank test. Cumulative TRGOO and OS after SEMS placement were calculated using a Kaplan–Meier analysis and compared using a log-rank test. TRGOO was censored when a patient died without RGOO or was alive with a patent stent, when a patient stopped consecutive hospital visit, or when a patient stopped oral intake without SEMS dysfunction. Factors associated with TRGOO were evaluated using a Cox hazard regression model. Given the small number of events with RGOO, only factors with *p* ≤ 0.10 in the univariate analyses were included in the multivariate analysis to avoid overfitting. A *p*-value < 0.05 was considered statistically significant. 

All statistical analyses were performed with EZR (Saitama Medical Center, Jichi Medical University, Saitama, Japan), which is a graphical user interface for R (The R Foundation for Statistical Computing, Vienna, Austria). More precisely, it is a modified version of R commander designed to add statistical functions frequently used in biostatistics [16]. 

## 3. Results

### 3.1. Patients

Between April 2006 and December 2022, a total of 95 cases with malignant GOO undergoing endoscopic second SEMS placement for RGOO were enrolled in this study. As shown in Table 1, patient characteristics were mostly well balanced between cSEMS (*n* = 48) and uSEMS (*n* = 47) groups, except for causes of the initial SEMS dysfunction. Major causes of the initial SEMS dysfunction were tumor ingrowth (52%) in the cSEMS group and stent migration (30%) and tumor ingrowth (26%) in the uSEMS group (*p* < 0.01). The chance to resume chemotherapy after second SEMS placement was similar between the groups (38% vs. 47%, *p* = 0.41).

### 3.2. Technical/Clinical Success and Adverse Events

Details of stent placement procedures are summarized in Table 2. Technical success was achieved in all cases with a median procedure times of 30 (IQR, 22–45) minutes in cSEMS and 37 (IQR, 29–51) minutes in uSEMS, respectively (*p* = 0.13). The clinical success rate was comparable between the groups (90% in cSEMS and 85% in uSEMS, *p* = 0.55). The median time to resume oral intake after second SEMS placement was 2 days in both groups (*p* = 0.70). The best GOOSS score after SEMS placement was also comparable between the two groups (*p* = 0.93); nearly three quarters of cases eventually reached a GOOSS score of three (77% in cSEMS vs. 74% in uSEMS, respectively).

**Figure 4:**
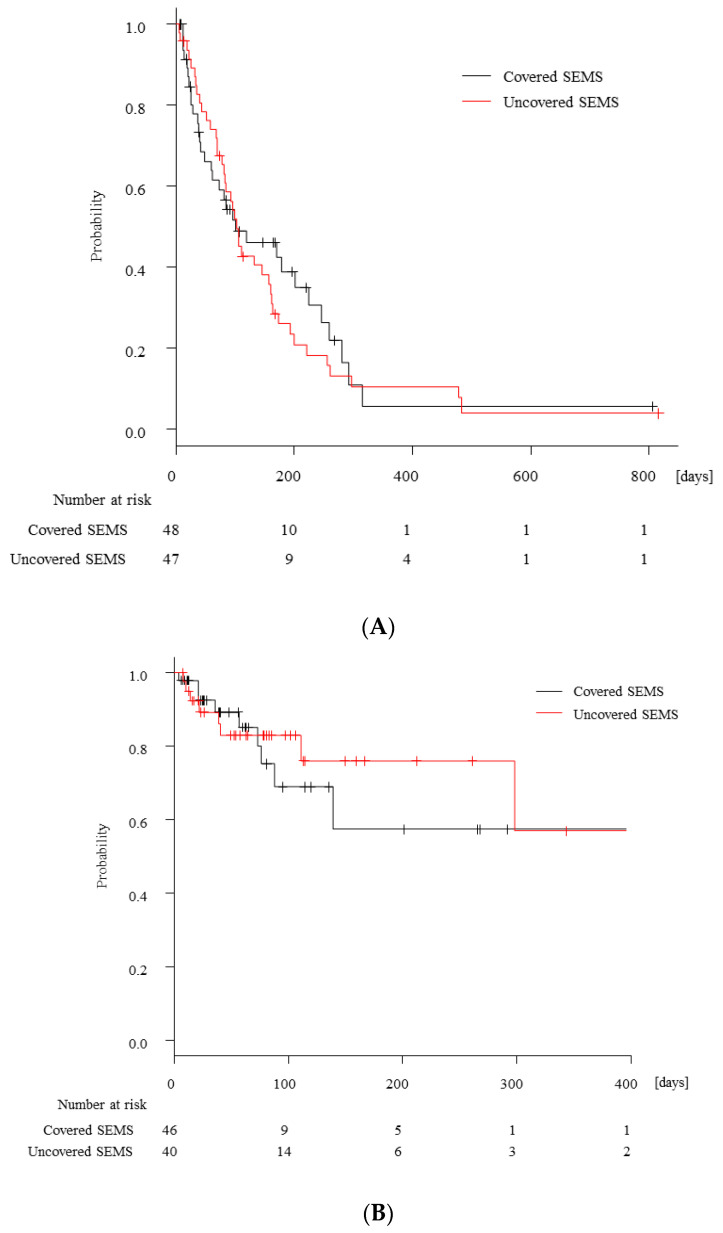

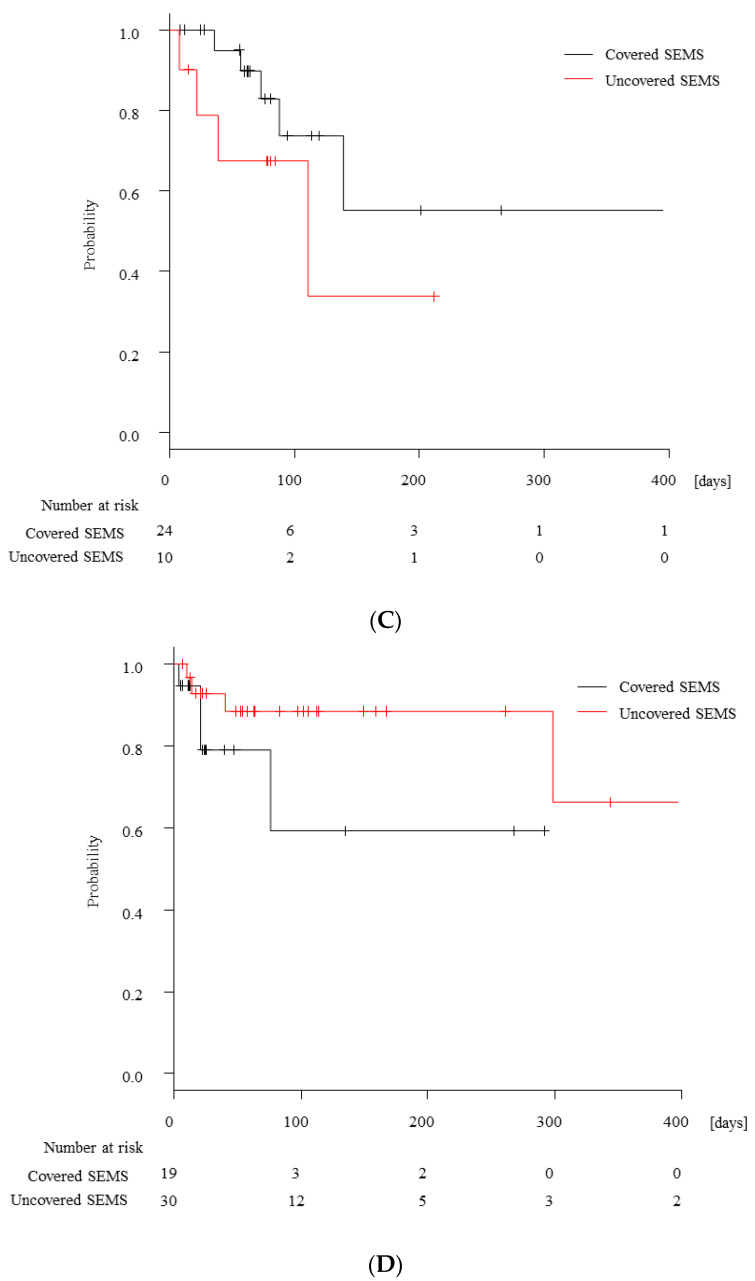
The Kaplan–Meier curves for overall survival and time to recurrent gastric outlet obstruction. (**A**) Kaplan–Meier estimates of overall survival after second self-expandable metal stent (SEMS) placement in cases with covered SEMS (cSEMS, black line) and uncovered SEMS (uSEMS, red line). (**B**) Kaplan–Meier estimates of time to recurrent gastric outlet obstruction after second SEMS placement in cases with cSEMS (black line) and uSEMS (red line). (**C**) Kaplan–Meier estimates of time to recurrent gastric outlet obstruction after second SEMS placement in cases whose initial SEMS had failed due to tumor ingrowth in cSEMS (black line) and uSEMS (red line). (**D**) Kaplan–Meier estimates of time to recurrent gastric outlet obstruction after second SEMS placement in cases whose initial SEMS had failed due to reasons other than tumor ingrowth in cSEMS (black line) and uSEMS (red line).

A total of 73 cases (77%) had expired during follow-up and had a median cumulative OS of 101 (95% confidence interval [CI], 60–224) days in cSEMS and 102 (95% CI, 78–157) days in uSEMS (*p* = 0.68, Figure 4A). Five cases had expired because of disease progression within 14 days of second SEMS placement, but there was no procedure-related mortality. The incidence of overall adverse events did not differ among the groups (15% vs. 17%, *p* = 0.78). The details of adverse events are shown in Table 3: perforation (6% in each group), bleeding (2% in only uSEMS), pancreatitis (4% in each group), cholangitis (4% in cSEMS and 2% in uSEMS), and aspiration pneumonia (1% only in uSEMS). Among six cases with perforation, four required surgery and two improved with supportive care alone. 

### 3.3. Recurrent Gastric Outlet Obstruction (RGOO) after Second SEMS Placement

The incidence of RGOO due to second SEMS dysfunction was comparable between the groups (19% vs. 13%, *p* = 0.58, Table 3). After second cSEMS placement, there was no case of tumor ingrowth, but stent migration occurred in one case. On the other hand, no migration but two cases of tumor ingrowth were observed in second uSEMS. Although the median TRGOO was not reached, with a median follow up of 57 days in cSEMS and 71 days in uSEMS, there was no statistical significance between the groups (*p* = 0.57, Figure 4B). And stent patency rates at 1, 2, and 3 months were comparable between the groups (60%, 47%, and 26%, respectively, in cSEMS vs. 70%, 55%, and 38%, respectively, in uSEMS). However, cumulative TRGOO tended to be longer in cSEMS when limited to cases undergoing second SEMS placement for tumor ingrowth (not reached vs. 111 days, *p* = 0.19, Figure 4C). On the contrary, uSEMS were more likely to have a longer cumulative TRGOO in cases undergoing second SEMS placement for RGOO other than tumor ingrowth (not reached vs. not reached, *p* = 0.12, Figure 4D).

The Cox regression analysis demonstrated chemotherapy after second SEMS placement was independently associated with longer TRGOO (the hazard ratio of 0.27, 95% CI, 0.08–0.93, *p* = 0.03). Additionally, Karnofsky performance status of <60 at second SEMS placement was a significant risk factor for shorter TRGOO (the hazard ratio of 3.07, 95% CI, 1.14–10.2, *p* = 0.04, Table 4).

## 4. Discussion

This retrospective study demonstrated that second SEMS placement was safe and effective for the management of RGOO regardless of the type of SEMS, in consistent with the existing literature [12,15,17,18,19,20]. The incidence of RGOO, cumulative TRGOO, and stent patency rates at 1, 2, and 3 months were comparable between cSEMS and uSEMS. However, cSEMS tended to have a longer TRGOO as compared to uSEMS when initial SEMS had been occluded by tumor ingrowth, while uSEMS were more likely to have longer TRGOO in cases who underwent second SEMS placement for other than tumor ingrowth. In addition, chemotherapy after second SEMS placement and Karnofsky performance status at second SEMS placement were independently associated with TRGOO.

About 25–35% of gastroduodenal SEMS for malignant GOO developed RGOO for some reason [12,17,18]. To prevent RGOO by improving initial SEMS function is obviously essential, but to optimize reintervention for RGOO is also important. Interestingly, a previous study demonstrated a combination of initial cSEMS followed by second uSEMS had a longer patency of second SEMS, as compared with other combinations of initial and second SEMS types [12]. However, it is more reasonable to select second SEMS according to the cause of initial SEMS rather than initial SEMS type. In our study, TRGOO of second SEMS was different between cSEMS and uSEMS according to the cause of initial SEMS dysfunction, though not statistically significant (Figure 4B,C). We speculated that second cSEMS is advantageous especially in the setting of RGOO due to tumor ingrowth since it can prevent tumor ingrowth and be less prone to migration when placed with stent-in-stent fashion because of more friction between the two SEMS. A recent study by Okamoto et al. reported a high migration rate of up to 20% in second cSEMS even after stent-in-stent placement [17], but all stents used in their study were cSEMS with a diameter of 20 mm. Meanwhile, 65% of cases underwent 24 mm cSEMS placement in our study. Thus, the frequent use of the large-bore cSEMS might be another reason for the better outcome in our study. On the other hand, uSEMS were more likely to have a longer TRGOO in cases who underwent second SEMS placement for other than tumor ingrowth (including tumor overgrowth, stent migration, inadequate stent expansion, and other reasons).

Our study demonstrated Karnofsky performance status and chemotherapy after second SEMS placement were independently associated with TRGOO but primary cancer (intrinsic vs. extrinsic cancer) and location of obstruction (Type II vs. others) were not. This finding indicates that the general condition as well as tumor burden rather than specific tumor conditions may have a greater impact on the outcomes of second SEMS since patients with recurrent GOO present with advanced tumors. Although chemotherapy was one of the contributing factors to TRGOO in our study, there are several conflicting reports about whether palliative chemotherapy contributes to an improved TRGOO or not [12,13,17,20]. This discrepancy may mainly arise from the differences in patient characteristics and chemotherapy; in other words, a various etiology of primary cancer as well as a variety of chemotherapeutic regimens among the studies. Therefore, to investigate the impact of chemotherapy on stent patency, it is necessary to consider anti-tumor effect by specific chemotherapy for each primary cancer.

Overall adverse events including procedure-related adverse events occurred in 15% and 17% of cases in cSEMS and uSEMS, respectively, which is similar to AEs of 20–25% with initial SEMS as well as second SEMS [8,9,12,15,17,19,20], suggesting the safety of second SEMS regardless of the SEMS type. However, we had six cases with gastroduodenal perforation in which a Wallflex stent was used in five out of six cases, at least as initial or second SEMS. Considering that axial force can be increased when two SEMSs were placed in stent-in-stent manner, caution is necessary to use a second SEMS with high axial force since it may cause mucosal damage at the end of the SEMS, especially in the tortuous portion of gastroduodenal tract. 

Some limitations of this study should be discussed here. Initially, this study was based on a nonrandomized retrospective design with a relatively small number of cases. Thus, there was imbalance in the patient characteristics especially in causes of the initial SEMS dysfunction. Secondly, various SEMSs with different mechanical properties were included in this study. Thirdly, the follow-up time was relatively short as cases with RGOO are generally associated with a poor prognosis, and late onset AEs might be underestimated. Finally, this study did not include cases undergoing endoscopic ultrasound-guided gastroenterostomy (EUS-GE), a novel alternative option to conventional intraluminal second SEMS placement in the salvage setting for RGOO [21,22,23]. Since a lumen-apposing metal stent does not traverse the tumor, EUS-GE may offer a higher chance to avoid SEMS dysfunction, especially due to tumor ingrowth. Therefore, prospective studies are warranted to investigate an optimal treatment approach for the management of RGOO. 

In conclusion, based on the results of our study, we concluded that second SEMS placement using cSEMS and uSEMS was similarly safe and effective for the management of RGOO. The type of second SEMS can be selected based on the cause of the initial SEMS dysfunction.

## Figures and Tables

**Table 1:** Patients’ characteristics.

	Covered SEMS (*n* = 48)	Uncovered SEMS (*n* = 47)	*p*-Value
Age, years	68 (61–77)	65 (58–72)	0.16
Sex, male	28 (58%)	29 (62%)	0.83
Primary cancer			0.08
Pancreas	29 (60%)	25 (53%)	
Stomach	11 (23%)	14 (30%)	
Biliary tract	2 (4%)	7 (15%)	
Others	6 (13%)	1 (2%)	
Karnofsky performance status			0.36
70–90	37 (77%)	32 (68%)	
30–60	11 (23%)	15 (32%)	
GOOSS score			0.37
0 (no oral intake)	28 (58%)	25 (53%)	
1 (liquids possible)	15 (31%)	12 (26%)	
2 (soft solids possible)	5 (10%)	10 (21%)	
Location of obstruction			0.82
Type I (proximal to the papilla)	15 (31%)	18 (38%)	
Type II (involving the papilla)	14 (29%)	10 (21%)	
Type III (distal to the papilla)	14 (29%)	13 (28%)	
Anastomosis	5 (10%)	6 (13%)	
Initial SEMS			0.75
Uncovered	25 (52%)	26 (55%)	
Covered	23 (48%)	21 (45%)	
Cause of initial SEMS dysfunction			<0.01
Ingrowth	25 (52%)	12 (26%)	
Overgrowth	11 (23%)	8 (17%)	
Migration	1 (2%)	14 (30%)	
Inadequate stent expansion	7 (15%)	4 (9%)	
Others (Kink/Stent fracture/Shortening, etc.)	4 (8%)	9 (19%)	
Time to initial SEMS dysfunction, days	75 (16–161)	34 (7–110)	0.13
Concomitant biliary obstruction	13 (27%)	14 (30%)	0.82
Peritoneal carcinomatosis	25 (52%)	21 (45%)	0.54
Ascites	26 (54%)	24 (51%)	0.84

GOOSS, gastric outlet obstruction scoring system; SEMS, self-expandable metal stent. All values are expressed as *n* (%) or median (interquartile range).

**Table 2:** Procedure details and efficacy of stent placement.

		Covered SEMS (*n* = 48)	Uncovered SEMS (*n* = 47)	*p*-Value
Stent product	Niti-S	-	29 (62%)	-
	Wallflex	-	18 (38%)	
	ComVi	48 (100%)	-	
Stent diameter, mm	20	17 (35%)	-	-
	22	-	47 (100%)	
	24	31 (65%)	-	
Stent length, cm	6	1 (2%)	9 (19%)	-
	8	4 (8%)	1 (2%)	
	9	3 (6%)	7 (15%)	
	10	4 (8%)	5 (11%)	
	12	28 (58%)	20 (43%)	
	15	6 (13%)	-	
	Multiple stenting	2 (4%)	5 (11%)	
Procedure time, min		30 (22–45)	37 (29–51)	0.13
Technical success		48 (100%)	47 (100%)	>0.99
Clinical success		43 (90%)	40 (85%)	0.55
Time to resume oral intake *, days		2 (2–3)	2 (2–3)	0.70
Best GOOSS score after SEMS placement				0.93
0 (no oral intake)		4 (8%)	5 (11%)	
1 (liquids possible)		1 (2%)	2 (4%)	
2 (soft solids possible)		6 (13%)	5 (11%)	
3 (low-residue or full diet possible)		37 (77%)	35 (74%)	
Chemotherapy after SEMS placement		18 (38%)	22 (47%)	0.41

GOOSS, gastric outlet obstruction scoring system; SEMS, self-expandable metal stent. * This outcome was evaluated only in patients who achieved clinical success after SEMS placement. All values are expressed as *n* (%) or median (interquartile range).

**Table 3:** Stent dysfunction and adverse events.

	Covered SEMS (*n* = 48)	Uncovered SEMS (*n* = 47)	*p*-Value
Stent dysfunction			
Overall	9 (19%)	5 (11%)	0.39
Stent migration	1 (2%)	0 (0%)	
Tumor ingrowth	0 (0%)	2 (4%)	
Tumor overgrowth	3 (6%)	1 (2%)	
Kinking	2 (4%)	1 (2%)	
Inadequate stent expansion	0 (0%)	0 (0%)	
Food impaction	2 (4%)	1 (2%)	
Stent fracture	1 (2%)	0 (0%)	
Reintervention			
Overall	9 (19%)	8 (17%)	0.82
Surgical procedure	1 (2%)	3 (6%)	
Endoscopic procedure	8 (17%)	5 (10%)	
Additional SEMS placement	6 (13%)	4 (8%)	
Food removal	2 (4%)	1 (2%)	
Adverse events			
Overall	7 (15%)	8 (17%)	0.78
Perforation	3 (6%)	3 (6%)	
Bleeding	0 (0%)	1 (2%)	
Pancreatitis	2 (4%)	2 (4%)	
Cholangitis	2 (4%)	1 (2%)	
Aspiration pneumonia	0 (0%)	1 (2%)	

cSEMS, covered self-expandable metal stent; uSEMS, uncovered self-expandable metal stent. All values are expressed as *n* (%).

**Table 4:** Factors associated with time to recurrent gastric outlet obstruction.

	Univariate		Multivariate	
	Hazard Ratio (95% CI)	*p*-Value	Hazard Ratio (95% CI)	*p*-Value
Age > 75 years	0.94 (0.27–3.32)	0.92		
Sex, male	0.37 (0.14–0.98)	0.04	0.66 (0.26–1.37)	0.29
Karnofsky performance status < 60	3.42 (1.27–9.22)	0.02	3.07 (1.14–10.2)	0.04
GOOSS score, 0	1.21 (0.46–3.17)	0.70		
Primary cancer, intrinsic cancers	1.24 (0.40–3.86)	0.72		
Peritoneal dissemination	1.06 (0.41–2.76)	0.91		
Ascites	1.52 (0.58–4.02)	0.39		
Location of obstruction, Type II	1.08 (0.38–3.08)	0.89		
Prior biliary drainage	0.55 (0.16–1.94)	0.33		
Initial SEMS type, covered SEMS	1.62 (0.62–4.27)	0.32		
Initial SEMS dysfunction within 2 weeks	1.08 (0.39–2.95)	0.88		
Cause of initial SEMS dysfunction, tumor ingrowth	1.46 (0.56–3.84)	0.44		
Second SEMS type, covered SEMS	1.32 (0.50–3.46)	0.57	1.71 (0.60–4.88)	0.31
Second SEMS diameter, 24 mm	1.01 (0.37–2.74)	0.98		
Second SEMS length > 12 cm	2.05 (0.70–5.97)	0.19		
Chemotherapy after second SEMS placement	0.19 (0.06–0.56)	<0.01	0.27 (0.08–0.93)	0.03

GOOSS, gastric outlet obstruction scoring system; SEMS, self-expandable metal stent; CI, confidence interval.

## Data Availability

Data is unavailable due to privacy or ethical restrictions.

## Abbreviations

GOO, gastric outlet obstruction; RGOO, recurrent gastric outlet obstruction; TRGOO, time to recurrent gastric outlet obstruction; AE, adverse event; OS, overall survival; cSEMS, covered self-expandable metal stent; uSEMS, uncovered self-expandable metal stent; GOOSS, gastric outlet obstruction scoring system; CI, confidence interval; and IQR, interquartile range.
